# COVID-19 Infection Complicated by Disseminated Intravascular Coagulation during Pregnancy—Two Cases Report

**DOI:** 10.3390/diagnostics12030655

**Published:** 2022-03-08

**Authors:** Małgorzata Skalska-Świstek, Hubert Huras, Andrzej Piotr Jaworowski, Rafał Świstek, Magdalena Kołak

**Affiliations:** 1 Department of Obstetrics and Perinatology, Medical College, Jagiellonian University, 23 Kopernika Str., 31-501 Krakow, Poland; hubert.huras@uj.edu.pl (H.H.); andrzej.jaworowski@uj.edu.pl (A.P.J.); magdalena.kolak@uj.edu.pl (M.K.); 2 Department of Anaesthesiology and Intensive Therapy, University Hospital in Krakow, 2 Jakubowskiego Str., 30-688 Krakow, Poland; rswistek@su.krakow.pl

**Keywords:** COVID-19, SARS-CoV-2, coagulopathies, disseminated intravascular coagulation syndrome, pregnancy, childbirth, DIC

## Abstract

Coagulopathies are one of the obstetric complications affecting the period of pregnancy, childbirth, and puerperium. One of the more severe and complex disorders of the haemostatic system is the disseminated intravascular coagulation syndrome (DIC), in which generalised activation of the coagulation system and activation of inflammatory cells occurs. DIC syndrome was observed in patients whose pregnancy was complicated by SARS-CoV-2 infection. Both the course of these cases and literature review indicate that particular notice should be paid to laboratory parameters of the coagulation system, closely monitoring the well-being of the foetus and, in the situation of acute DIC development, it is advised to deliver a baby and initiate intensive therapy.

## 1. Introduction

Obstetric coagulopathies are disturbances in the haemostatic system that undoubtedly affect the period of pregnancy, childbirth, and puerperium. They can be associated with impaired clot formation and bleeding tendency, as well as increased blood clotting. One of the complex obstetric coagulopathies is the syndrome of disseminated intravascular coagulation.

Disseminated intravascular coagulation syndrome (DIC) is secondary to many clinical conditions (multi-organ trauma, sepsis, septic shock), such as obstetric conditions (intrauterine foetal death, amniotic fluid embolism, placental abruption, eclampsia). Its essence is the generalised activation of blood coagulation with the production of a large amount of fibrin, which binds platelets and forms blood clots, ultimately leading to consumption coagulopathy [1].

Even an uneventful pregnancy triggers numerous changes in the haemostatic system. These are expressed by an increase in the concentration of pro-thrombotic factors with simultaneous decrease in plasma fibrinolytic activity. Changes in the coagulation system most often occur in the third trimester of pregnancy, when, among others, the increase of a coagulation factors VII, VIII, X, tissue plasminogen activator (t-PA), urokinase plasminogen activator (u-PA), and simultaneous decrease of plasma fibrinolytic activity are observed, leading to increased D-dimer concentration, which is the indicator of a clot formation. However, there are no established reference ranges for the concentration of coagulation factors and fibrinolysis processes in pregnant women. In everyday practice, tests assessing the concentration of D-dimers, fibrinogen, prothrombin time (PT), activated partial thromboplastin time (APTT), and peripheral blood count are used [2,3].

DIC can be an acute or a chronic process. Acute DIC progresses very rapidly, it is often accompanied by bleeding (from postoperative wounds, genital tract, oral cavity) and multiorgan ischaemia, leading to impaired function of important organs: renal failure, hepatic failure, and impaired gas exchange in the lungs. Moreover, an ischaemic or haemorrhagic stroke is also possible. In acute DIC, there is an uncontrolled breakdown of the compensatory mechanisms and consumption coagulopathy. The most common causes of acute DIC include sepsis, extensive injuries, post-transfusion reaction, obstetric complications. Chronic DIC is usually mild and is associated with minor signs of bleeding disorders: easy bruising, recurrent nosebleeds, skin ecchymosis. It is most often observed in people with malignant tumours, and in obstetrics patients in the case of intrauterine foetal death [4,5].

The pathogenesis of DIC development is difficult to be clearly defined. Among obstetric causes, the most common include placental abruption, postpartum haemorrhage, severe preeclampsia, amniotic fluid embolism, acute pregnancy fatty liver, intrauterine foetal death, infection (septic miscarriage, intrauterine infection), or SARS-CoV-2 infection.

The SARS-CoV-2 virus enters endothelial cells by binding the protein S with the angiotensin converting enzyme type 2 (ACE2) [6]. An inflammatory process of the endothelium develops, which damages its cells and disrupts its anticoagulant function, increasing the risk of thrombosis [7]. The result is a high level of von Willebrand factor (vWF) [8]. Concomitant disorders within the vWF-ADAMTS13 axis, associated with the activation of platelets and the coagulation cascade, intensify coagulopathy, and increase the risk of blood clots [9]. In addition, binding the virus with ACE2 inhibits the expression of ACE2, which may weaken the anti-inflammatory and anticoagulant ACE2-angiotensin-MAS receptor axis and enhance the pro-inflammatory and prothrombotic effects of angiotensin II [10]. The interaction between activated innate response mechanisms (neutrophils, monocytes, cytokines that they release), the coagulation system (platelets, coagulation cascade), and the complement system triggers a process similar to immune thrombosis resulting in the development of thromboembolism and the formation of blood clots in large and small vessels [11]. Similar processes are observed in other complications of pregnancy associated with an increased risk of thromboembolism, such as HELLP syndrome (haemolysis, elevated liver enzymes, and low platelets), acute fatty liver of pregnancy, thrombotic thrombocytopaenic purpura, pregnancy-related haemolytic–uremic syndrome, and systemic lupus erythematosus. The risk of thrombotic events is increased by coagulopathy accompanying COVID-19, in the course of which high concentrations of D-dimers, fibrinogen degradation products, fibrinogen, thrombocytopaenia, changes in activated partial thromboplastin time (APTT) and prothrombin time (PT, prothrombin time) are observed, as well as increased stability of the blood clot [12].

Fan et al. reviewed the literature on maternal–foetal complications related to SARS-CoV-2. The study showed that pregnant women had worse clinical course and poorer outcome compared to non-pregnant controls. Additionally, they analysed complications that occurred during pregnancy. They showed that premature rupture of membranes (PROM), preterm labour, foetal growth restriction (FGR), intrauterine foetal demise, and neonatal death are more frequently observed in pregnant patients with SARS-CoV-2 infection [13].

The aim of this study is to describe the clinical cases of two pregnant patients who developed intravascular coagulation syndrome with the coexisting SARS-CoV-2 infection.

## 2. Case 1 Presentation

A 25-year-old patient, gravida 1 para 1, in the 34th week of gestation, was admitted to the Department of Obstetrics and Perinatology of the Jagiellonian University Hospital in Krakow in April 2021 due to a reduced feeling of foetal movements during the last 24 h and the feeling of irregular contractions. The patient was taking medications due to hypothyroidism diagnosed in the first trimester of pregnancy (currently clinically euthyroid). Sanitary regime was applied because the patient had a coexisting SARS-CoV-2 virus infection. The infection was confirmed by a PCR test on the previous day. On admission to the hospital, the body temperature was 36.2 °C. Other vital signs: regular HR 90 bpm, BP 130/70 mmHg, oxygen saturation 98%. The patient reported fever between 39 and 40 °C and headache without symptoms typical for SARS-CoV-2 infection, such as taste and smell disorders, dyspnoea, cough, runny nose, muscle pain, sore throat. She was taking paracetamol. The patient also voluntarily did not receive any dose of the COVID-19 vaccine.

On initial examination per vaginum: the vaginal part of the cervix was slightly shortened, directed posteriorly, and the opening was 0.5 cm (Bishop score of 2). Uterine muscle tension was normal with irregular, weak contractions. There was no active vaginal bleeding or leakage of amniotic fluid during the examination. Discharge from the vagina was blood-stained. The ultrasound examination revealed a living foetus in the cephalic position, with the present heart rate of about 160 bpm. The estimated foetal weight was 2400 g, the foetal placenta was located on the anterior wall of the uterine cavity. Foetal doppler flows were normal: umbilical artery pulsation index UA-PI 0.86, pulsation index in the middle cerebral artery MCA-PI 1.29. Amniotic fluid index AFI = 12 cm.

Laboratory tests were performed (Table 1). Main attention was drawn to the result of D-dimers, fibrinogen, AST, ALT, LDH. The analysis of the above parameters revealed DIC and clinical signs of the HELLP (haemolysis, elevated liver enzymes, and low platelets) syndrome.

**Figure 1:**
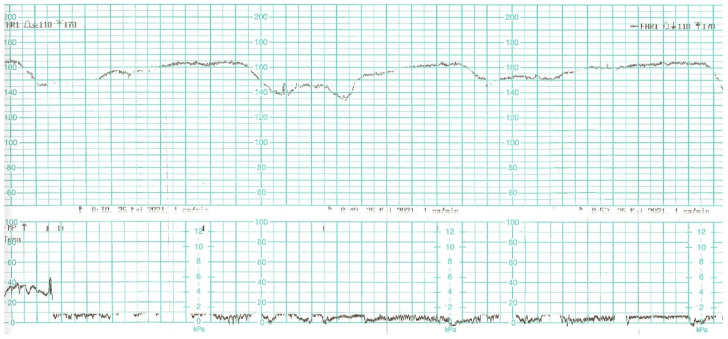
Cardiotocography recording in a patient 1—normal baseline, reduced variability.

While waiting for the results of laboratory tests, a control cardiotocographic (CTG) recording was made. During the CTG recording, the foetal heart rate was found between 140 and 160 bpm. Within approximately 25 min of recording, reduced short-term variability with single decelerations and no acceleration was found—a pathological record that may suggest foetal hypoxia (Figure 1).

In view of the above-mentioned test results, it was decided to perform an emergency caesarean section. Indications for the procedure were intrauterine asphyxia, acute DIC and suspicion of HELLP syndrome. The surgery was performed in a sanitary regime. The anaesthesiologist used general anaesthesia due to the disturbances in the coagulation system and the urgency of the procedure. During the operation, the patient received 1 g of tranexamic acid and 3 g of fibrinogen, 2 units of fresh-frozen plasma and 2 units of concentrated platelets. Pelvic drainage was used to better control possible bleeding into the abdominal cavity in the early postoperative period. Broad-spectrum antibiotic therapy was ordered.

The newborn’s Apgar score of 0 was recorded in the 1st, 3rd, and 5th minute. After childbirth, the baby was in an extremely poor condition, without heart activity, hypotonic, unresponsive. Cyanosis was found on the peripheral parts of the body. Cardiopulmonary resuscitation according to ERC guidelines was started. Initially, the child was ventilated using a mask, then it was intubated. After approximately 15 min of CPR, first foetal heartbeats were obtained. The intubated and mechanically ventilated child was transported to the Neonatal Intensive Care Unit in a sanitary regime. The newborn was isolated until receiving a negative result for COVID-19.

**Figure 2:**
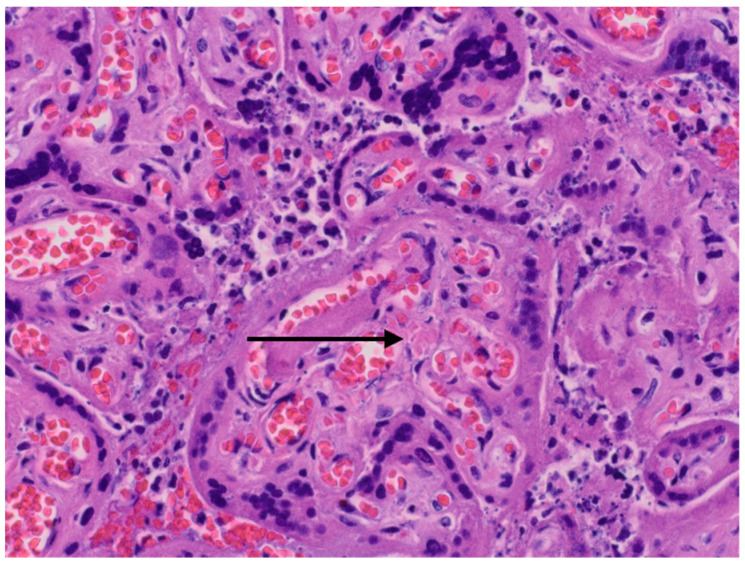
Histopatological images. Arrow—small infarcts.

**Figure 3:**
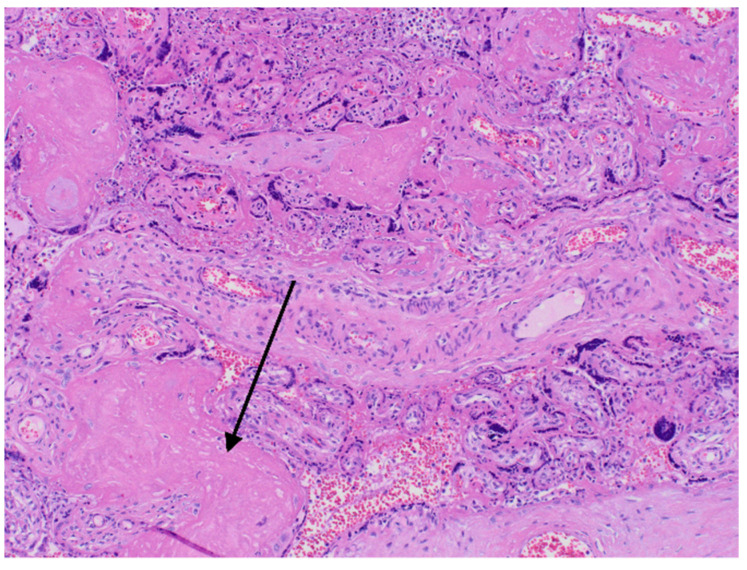
Histopatological images. Arrow—fibrin deposits.

The results of neonatal examinations are summarised in Table 2. Significant changes of D-dimers, fibrinogen, AST were observed. Arterial blood gas test showed: pH 6.618 N: 7.35–7.45), pO_2_ 64.9 mmHg (N: 50–70), pCO_2_ 71.8 mmHg (N: 45–55), cLac-30 mmol/L (N: <2). The results indicated a significant metabolic acidosis, chronic hypoxia and coagulation disorders. Brain ultrasound revealed increased diffuse echogenicity of brain tissue, scanty, high-resistance blood flow. Abdominal ultrasound showed abnormal renal and visceral blood flow. At the same time, anuria, hypoglycaemia and hypotension persisted. The newborn did not respond to the treatment and succumbed 24 h after birth. In the postmortem examination congestion of the entire brain, especially the choroid plexus, the central area around the ventricles, and the meninges was found. The image suggested blockage of the venous outflow. Early, diffuse ischaemic changes with the necrosis in the cerebral cortex, as well as blood clots in small vessels in the brain were visualised. The newborn’s lungs were also congested, with foci of atelectasis. The postpartum histopathology examination revealed a strongly hyperaemic placenta with small infarcts and abundant fibrin deposits (chronic intervillositis) (Figure 2 and Figure 3).

**Figure 4:**
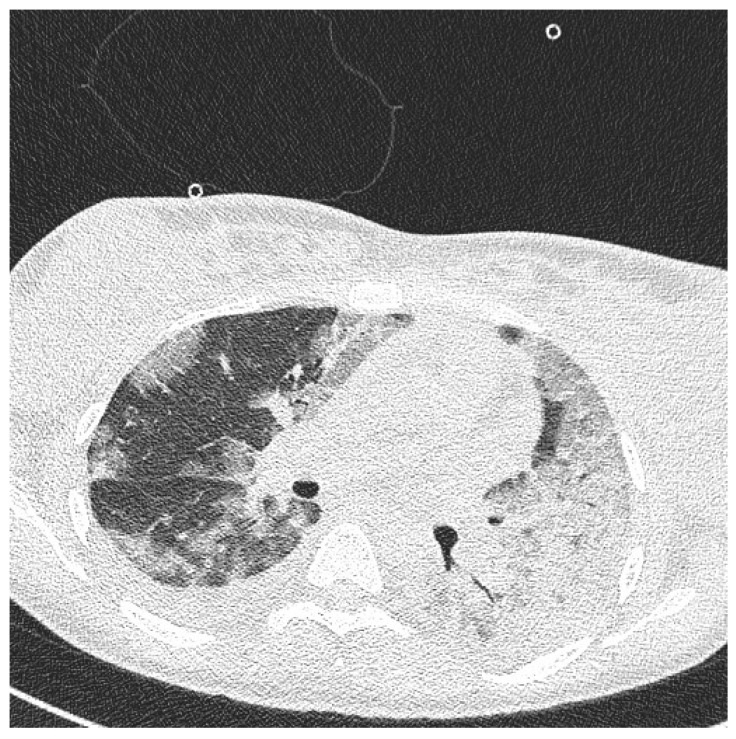
Chest Computed Tomography (HRCT).

**Figure 5:**
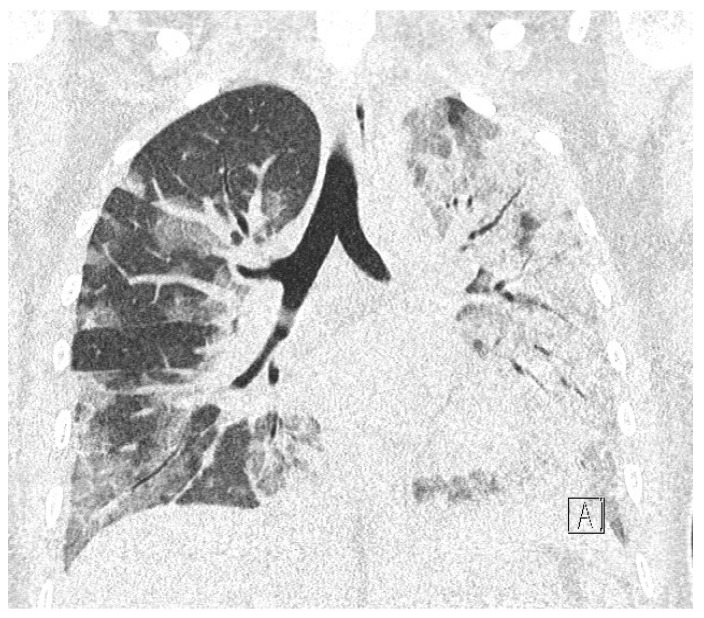
Chest Computed Tomography (HRCT).

On the day of caesarean section, the patient’s vital parameters were: BP 130/80 mmHg, HR 110 bpm, Sat. 96%, breaths 20/min. Laboratory tests showed persistent abnormal clotting parameters. The drainage from the pelvis was 800 mL of blood, and in the control abdominal ultrasound examination the subcutaneous haematoma was observed. Due to the above, the patient was qualified to the relaparotomy and pelvic revision. During the procedure, another 2 g of fibrinogen, 2 units of platelet concentrate and 2 units of packed red blood cells were given. During the next two days, the patient was monitored in a postoperative room. Stable laboratory test values were obtained. Table 1 summarises the results of the patient’s examinations. A CT scan of the chest was performed. Massive converging zones of bubble densities in the type of frosted glass and paving stones, suggesting viral inflammation (typical for SARS-CoV-2 infection) were visible—see Figure 4 and Figure 5. Due to the gradually increasing respiratory failure (saturation 90%, and hypoxia—pO2 54 mmHg in a control arterial probe), high-flow nasal oxygen therapy was started. The satisfactory effect was not achieved. Sedation, intubation, and mechanical ventilation was initiated. After initial stabilisation the patient was transferred to the Intensive Care Unit, where she stayed for 8 days until improvement in oxygenation was achieved. She was successfully extubated and transferred to the Maternity Ward, from where she was discharged on the 22nd day of hospitalisation in good general condition.

Long-term follow-up shows the patient was in good physical condition but she was still receiving psychological assistance.

## 3. Case 2 Presentation

A 36-year-old patient, gravida 5 para 4, in the 35th week of pregnancy, was admitted to the Department of Obstetrics and Perinatology of the University Hospital in Krakow in April 2021 due to general weakness, muscle pain and dyspnoea. The patient reported symptoms typical for SARS-CoV-2 infection, such as: cough, runny nose, headache, anosmia and ageusia for two days. Other than that, the course of pregnancy was uneventful so far, she took levothyroxine due to hypothyroidism diagnosed in the first trimester of pregnancy (currently clinically euthyroid). On admission, the patient’s condition was assessed as good, the patient was cardiovascularly and respiratorily stable: BP 120/60 mmHg, HR 84 bpm, body temperature 36.3 °C, saturation 98% in the atmospheric air. On admission, physical, ultrasound, laboratory, and microbiological examinations were performed. Due to the nature of the symptoms reported by the patient a nasopharyngeal swab was taken. The SARS-CoV-2 infection was confirmed. The patient was admitted to the ward with an appropriate sanitary regime. Gynaecological examination was performed and did not present any abnormalities (Bishop score of 2, normal uterine tone). The ultrasound examination revealed a living foetus in the cephalic position (FHR 140 bpm). The estimated foetal weight was 2646 g, the placenta at the fundus of the uterine cavity. Foetal doppler flows: umbilical artery pulsation index UA-PI 0.90, pulsation index in the middle cerebral artery MCA-PI 1.80. Amniotic fluid index AFI = 13 cm.

**Figure 6:**
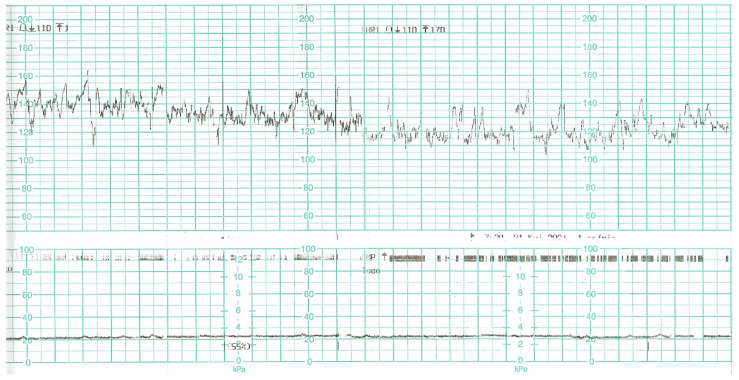
Cardiotocographic record made on day 0 of hospitalisation-normal baseline, normal variability.

The control CTG record was normal, revealing normal cardiac activity and reactive variability (Figure 6). Laboratory tests are summarised in Table 3. Due to the symptoms of upper respiratory tract infection and elevated values of inflammatory parameters (CRP 52.50 mg/L [N: <5]), prophylactic antibiotic therapy with third-generation cephalosporin was ordered. The patient was also prescribed a prophylactic dose of low-molecular-weight heparin.

**Figure 7:**
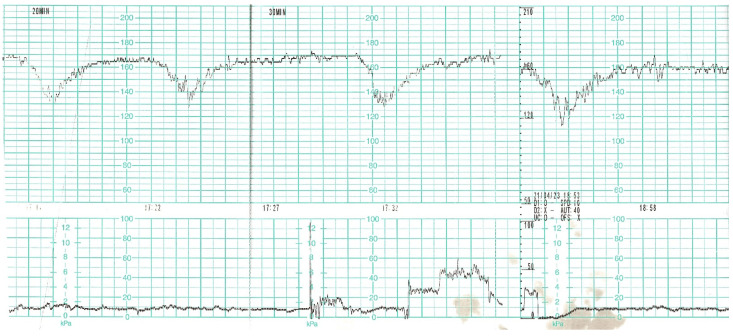
CTG record on day 2 of hospitalisation. Tachycardia, decelerations.

During the next two days of hospitalisation, the patient’s general condition was stable. However, in the controlled laboratory test disturbingly high concentration of D-dimer, decreased platelet level and fibrinogen values were observed (Table 3). The differential diagnosis included: haemostatic disorders in the course of COVID-19 infection, the onset of DIC. In the follow-up cardiotocographic record, tachycardia 170 bpm, minimal variability and recurrent decelerations were observed (Figure 7). Due to the development of DIC and the intrauterine foetal asphyxia, the patient was qualified for an emergency caesarean section. Before the procedure, the patient received 1 unit of platelet concentrate. The surgery was uneventful. The pelvic drainage comprised 100 mL of serous blood; therefore, it was removed on the first postoperative day. Due to anaemia (HGB 8.7%, HCT 26%) as well as weakness and dizziness reported by the patient, 2 units of packed red blood cells were transfused, which resulted in normalisation of the red blood cell system parameters.

The male newborn was born in an average general condition. The baby’s Apgar score was 6/7/10 points in the 1st, 3rd, and 5th minute of life, respectively. The child’s laboratory tests were within the normal range. Nasopharyngeal swab for COVID-19 infection was negative, while the result of the bronchoalveolar swab showed the presence of SARS-CoV-2 virus. The histopathology examination of the placenta showed the presence of small foci of inflammatory infiltrates composed of neutrophils among the villi of the placenta. The patient and the newborn were isolated for 9 days, after which they were discharged home in good general condition. One month after leaving the hospital, the mother and the infant came for a check-up at the neonatal clinic. The infant was breastfed and gained weight accordingly. His condition was declared to be good. His mother was in good condition too.

## 4. Discussion

In the last 2 years of the COVID-19 pandemic, it has been noticed that SARS-CoV-2 infection increases the pro-thrombotic potential. Laboratory picture of haemostasis parameters in a pregnant patient suffering from COVID-19, especially those with acute course, may resemble DIC, SIC (sepsis-induced coagulopathy), TMA (thrombotic microangiopathy) and HELLP syndrome [14].

The occurrence of acute DIC syndrome requires differential diagnosis. Endothelial damage results in the appearance of thrombotic microangiopathy, which accompanies other severe complications of pregnancy such as: HELLP syndrome (haemolysis, elevated liver enzymes, and low platelets), acute fatty liver of pregnancy, thrombotic thrombocytopaenic purpura, pregnancy-related haemolytic–uremic syndrome, and systemic lupus erythematosus). Symptoms and abnormalities in laboratory test results in these syndromes may help to make a diagnosis, but this is not always possible due to the overlapping symptoms (Table 4).

The course of SARS-CoV-2 infection in most pregnant women is mildly symptomatic. There is no doubt, however, that both COVID-19 and pregnancy increase the prothrombotic potential [16]. Thrombocytopaenia, hypofibrinogenaemia, prolonged prothrombin time, prolonged partial thromboplastin time after activation, an increase in the level of fibrinogen degradation product (FDP), and D-dimers may accompany both SARS-CoV-2 infection and DIC syndrome. However, in the DIC syndrome the D-dimers level significantly exceeds the normal values.

Repeated laboratory tests and the analysis of their results at intervals are great diagnostic tools. Such approach allows to detect abnormalities in the coagulation system and implement treatment in advance. The evaluation of the above-mentioned parameters was used to create an algorithm to assess the risk of coagulopathy in the course of COVID-19 [17]. 

A modified DIC risk assessment scale was developed for pregnant women—ISTH DIC score (Table 5). The score of ≥26 points suggests a high probability of DIC. The scale is characterised by a high percentage of sensitivity (81%) and specificity (96%) for the diagnosis of intravascular coagulation during pregnancy [15]. In both of the above-described cases, the patients were highly likely to develop the intravascular coagulation syndrome. In the case of the first patient, the total of points was 52 and in the case of the second patient it was 27 points directly before caesarean section. The scale was used retrospectively in our cases, as so far it has not been implemented in our ward. Our cases confirm that the algorithm has high sensitivity. It is worth adapting the scale to daily clinical practice. 

Previous research has shown that the so-called cytokine storm caused by abnormal, excessive activation of the immune system after SARS-CoV-2 infection leads in some cases to a sudden deterioration of the patient’s health. In such a situation, a large amount of cytokines and chemokines are secreted, for example TNF-α, IL-1, IL-6, IL-8, which stimulate other cells of the immune system and the formation of a large inflammation focus, and, as a result, micro- and macro-vascular thrombosis leading to organ failure [14].

Recently, Servante presented a literature review on thromboembolic complications in pregnant patients with SARS-CoV-2 infection. One thousand and sixty-three patients participated in the study. Seventeen patients passed away. Among them, two deaths were due to the development of DIC. There was also a higher risk of developing thromboembolic complications. In two patients pulmonary embolism was reported, with concurrent basilar artery thrombosis in one case. The condition of 132 patients was serious and required admission to the Intensive Care Unit [18]. Our cases were complicated by a neonatal death in the first case. In long-term follow-up, both mothers and the second newborn were in good condition.

The use of antithrombotic prophylaxis in a pregnant patient with coexisting COVID-19 infection seems to be justified. Heparin reduces the prothrombotic potential accompanying a systemic inflammatory reaction, for example in the acute course of COVID-19. However, in the case of acute DIC with severe haemorrhagic diathesis, administration of heparin is contraindicated. The only exception is when the thrombotic processes outweigh the possible risk of bleeding. The anticoagulant and anti-inflammatory properties can therefore improve the prognosis, provided that their use is currently safe for both the pregnant woman and the foetus [19,20]. In our cases, the second patient received low-molecular-weight heparin (LWMH) prophylaxis, the first one was operated on a day of admission so there was no time to administer heparin before the operation. On the other side, Jevtic et al. conducted a survey study which suggested that COVID-19-associated coagulopathy (CAC) was uncommon in pregnancy. What is more important, venous thromboembolisms were observed in some pregnant patients despite thromboprophylaxis [21]. 

Furthermore, thyroid hormone levels can influence the coagulation system. Some studies have shown that high levels of thyroid hormone decrease the risk of bleeding and intensify coagulation [22]. Both patients were taking medications due to hypothyroidism diagnosed in the first trimester of pregnancy and they were clinically euthyroid during the infection.

Tranexamic acid (TXA) is an antifibrinolytic agent. It acts by stabilising clot formation which is often reduced by systemic fibrinolysis. The WOMAN study has shown that TXA significantly reduces the risk of postpartum bleeding and maternal mortality [23]. Use of TXA during pregnancy complicated by intravascular coagulation is still controversial [24]. It must be used with extreme caution. The use of TXA may be indicated in DIC with enhanced fibrinolysis and severe haemorrhage [25]. In our first case, the patient received TXA only once-during caesarean section due to abnormal bleeding and suspected beginning of fibrinolysis.

The severe course of SARS-CoV-2 infection in pregnancy increases the prothrombotic potential also in the foetal part of the placenta. Postpartum histopathology tests showed the presence of infarction foci and inflammatory infiltrates. Recently, a meta-analysis was published comparing the histopathology examinations of the placenta of pregnant patients with high-risk pregnancy and the placenta of patients with pregnancy complicated by COVID-19 infection. The meta-analysis showed a significant reduction in vascular perfusion, foci of acute and chronic inflammation, foci of thrombosis, areas of fibrin deposits [26,27]. This means that the risk of stillbirth by the COVID positive patient is much higher than in the general population. Similar changes were described in the histopathology examination of the placenta of the presented patients.

Indirectly, the deterioration of the condition of the foetuses in the above-described cases could be proved by pathological CTGs which were the indication to perform caesarean section. The general condition of the newborns after birth was assessed as severe or moderately severe.

Januszewski et al. compared the results of studies in pregnant women with complications triggered by the SARS-CoV-2 infection. The researchers showed that most of the pregnant patients required hospitalisation during the third trimester of pregnancy. Therefore, it concluded how important it is to vaccinate prior to the 30th week of pregnancy. This may reduce the risk of severe course of the disease [28]. In our both cases, the patients did not receive vaccination, because at that time it was not yet recommended and was not available for pregnant women in our country.

Various studies led to different conclusions regarding the course of COVID-19 in pregnant women. They had worse, milder or similar course of COVID-19 disease compared to non-pregnant controls [21,29]. It might be caused by different time of conducted studies and diverse variants of the virus (nowadays the Omicron variant is prevalent), the vaccination percentage of the population, the virulence of the pathogen, the recurrence. The cases presented above concern older variants of the SARS-CoV-2 virus whose courses were more severe than observed in our clinic right now. Further research in that field is required.

DIC is a disease that has been known well; however, the coincidence between the DIC and recent epidemic disease COVID-19 as a risk factor is still not fully recognised. Actual knowledge stands that SARS-CoV-2 virus will not disappear, and the COVID-19 disease will occur more or less frequently during the seasons [30]. These case reports are intended to draw attention to the inclusion of co-occurrence of COVID-19 in the diagnosis of DIC in pregnancy. DIC is a serious complication that is potentially fatal for mother and foetus.

Having experience from the mentioned cases the consecutive aim of our work is to estimate risk factors, preceding symptoms, and laboratory findings, as well as validation of the DIC risk assessment scale in pregnancy with COVID-19 coinfection. We assume that in future, our work will help to reveal potential danger in advance to ensure safety of women and their children.

## 5. Conclusions

Presented clinical cases showed how important it is to perform a proper and wide diagnostic procedure, including laboratory tests, imaging, and foetus condition monitoring with correct interpretation of the results in relation to the general condition of the pregnant patient to establish an accurate, effective and multidisciplinary treatment. It is worth noting that acute DIC can develop suddenly, leading to serious and life-threatening complications for both the pregnant woman and the foetus. For daily medical practice, when caring for a pregnant patient with SARS-CoV-2 infection, attention should be paid not only to the severity of the respiratory involvement, but also to the laboratory parameters of the coagulation system, in particular the level of fibrinogen, which may be crucial for the early diagnosis of developing DIC.

## Figures and Tables

**Table 1:** Summary of the results of the patient’s tests at the time of admission to the hospital (before caesarean section) and on the first postoperative day.

Test	The Day of Admission to the Hospital, before Caesarean Section	First Postoperative Day	Reference Range
WBC	10.80 × 10^3^/uL	13.17 × 10^3^/uL	(4.0–10.0)
RBC	4.96 × 10^6^/uL	2.90 × 10^6^/uL	(4.0–5.0)
HGB	15.2 g/dL	8.9 g/dL	(12.0–16.0)
HCT	43.7%	24.6%	(37.0–47.0)
PLT	71 × 10^3^/uL	85 × 10^3^/uL	(140–440)
PT	13.8 s	11.5 s	(12–16)
INR	1.29	1.05	(0.9–1.20)
APTT	59.6 s	38.9 s	(26–36)
Fibrinogen	no clot	3.00 g/L	(1.8–3.5)
D-dimers	>34.47 mg/L FEU	1.33 mg/L FEU	(<0.55)
AST	201 U/L	85 U/L	(10–35)
ALT	76 U/L	42 U/L	(10–35)
LDH	1292 U/L	474 U/L	(135–214)

**Table 2:** The newborn’s examination results.

Test	The Newborn’s Examination Results	Reference Range
WBC	29.23 × 10^3^/uL	(10.0–26.0)
RBC	4.78 × 10^6^/uL	(4.4–5.9)
HGB	17.3 g/dL	(16.0–22.0)
HCT	58.3%	(54–66)
PLT	107 × 10^3^/uL	(150–450)
PT	51.1 s	(12–16)
INR	6.92	(0.9–1.20)
APTT	no clot	(26–36)
Fibrinogen	no clot	(1.8–3.5)
D-dimers	>34.47 mg/L FEU	(<0.55)
AST	3007 U/L	(10–35)
ALT	404 U/L	(10–35)

**Table 3:** Laboratory test results on the day of admission to hospital and second day of hospitalisation.

Test	Day of Admission	Second Day of Hospitalisation	1st Day after Caesarean Section	Reference Range
WBC	6.68 × 10^3^/uL	5.91 × 10^3^/uL	13.84 × 10^3^/uL	(4.0–10.0)
RBC	4.23 × 10^6^/uL	4.31 × 10^6^/uL	3.27 × 10^6^/uL	(4.0–5.0)
HGB	13.4 g/dL	13.6 g/dL	10.5 g/dL	(12.0–16.0)
HCT	39.8%	39.7%	30.0%	(37.0–47.0)
PLT	122 × 10^3^/uL	51 × 10^3^/uL	76 × 10^3^/uL	(140–440)
PT	10.1 s	12.8 s	10.7 s	(12–16)
INR	0.91	1.19	0.97	(0.9–1.20)
APTT	26.7 s	44.1 s	28.2 s	(26–36)
Fibrinogen	3.1 g/L	0.65 g/L	2.01 g/L	(1.8–3.5)
D-dimers	2.46 mg/L FEU	>34.47 mg/L FEU	1.89 mg/L FEU	(<0.55)
AST	23 U/L	88 U/L	61 U/L	(10–35)
ALT	15 U/L	23 U/L	25 U/L	(10–35)
LDH	223 U/L	621 U/L	-	(135–214)

**Table 4:** Laboratory findings typical for pregnancy complications in which DIC may occur [15].

Laboratory Findings	HELLP Syndrome	AFLP	TTP	HUS	Exacerbation of SLE
Thrombocytopaenia (<100 thou./mm^3^)	More than 20,000	More than 50,000	20,000 or less	More than 20,000	More than 20,000
Haemolysis (%)	50–100	15–20	100	100	14–23 with APA
Anaemia (%)	Less than 50	Absent	100	100	14–23 with APA
DIC (%)	Less than 20	50–100	Rare	Rare	Rare
Hypoglycaemia (%)	Absent	50–100	Absent	Absent	Absent
VW factor multimers (%)	Absent	Absent	80–90	80	Less than 10
ADAMTS13 less than 5% (%)	Absent	Absent	33–100	Rare	Rare
Impaired renal function (%)	50	90–100	30	100	40–80
LDH (IU/L)	600 or more	Variable	More than 1000	More than 1000	with APA
Elevated ammonia (%)	Rare	50	Absent	Absent	Absent
Elevated bilirubin (%)	50–60	100	100	NA	Less than 10
Elevated transaminases (%)	100	100	Usually mild *	Usually mild *	with APA

Abbreviation: HELLP: haemolysis, elevated liver enzymes, low platelets; AFLP: acute fatty liver of pregnancy; TTP: thrombotic thrombocytopaenic purpura; HUS: haemolytic uremic syndrome; SLE: systemic lupus erythematosus; APA: antiphospholipid antibodies with or without catastrophic antiphospholipid syndrome; DIC: disseminated intravascular coagulopathy; VW: von Willebrand; ADAMTS: von Willebrand factor-cleaving metalloprotease; LDH: lactic dehydrogenase; NA: values are not available. * Levels less than 100 international units/L.

**Table 5:** DIC risk assessment scale in pregnancy according to Erez et al. (2014) [17].

PLT [thou./mm^3^]	>185 = 0 point 100–185 = 1 point 50–100 = 2 point <50 = 1 point
PT (PTp-PTn)	<0.5 = 0 point 0.5–1.0 = 5 point 1.0–1.5 = 12 point >1.5 = 25 point
Fibrinogen [g/L]	<3.0 = 25 point 3.0–4.0 = 6 point 4.0–4.5 =1 point >4.5 = 0 point
total points	≥26 points—high probability of DIC

Abbreviations: PLT (plates)—platelet count, PT (prothrombin time), PTp—patient’s PT, PTn—laboratory standard (lower limit).
